# Intermediate Levels of Network Heterogeneity Provide the Best Evolutionary Outcomes

**DOI:** 10.1038/s41598-017-15555-7

**Published:** 2017-11-10

**Authors:** Flávio L. Pinheiro, Dominik Hartmann

**Affiliations:** 1 Collective Learning Group, The MIT Media Lab, Massachusetts Institute of Technology, Cambridge, MA USA; 2 Chair for Innovation Management and Innovation Economics, University of Leipzig, Leipzig, Germany; 3 Fraunhofer Center for International Management and Knowledge Economy, Leipzig, Germany

## Abstract

Complex networks impact the diffusion of ideas and innovations, the formation of opinions, and the evolution of cooperative behavior. In this context, heterogeneous structures have been shown to generate a coordination-like dynamics that drives a population towards a monomorphic state. In contrast, homogeneous networks tend to result in a stable co-existence of multiple traits in the population. These conclusions have been reached through the analysis of networks with either very high or very low levels of degree heterogeneity. In this paper, we use methods from Evolutionary Game Theory to explore how different levels of degree heterogeneity impact the fate of cooperation in structured populations whose individuals face the Prisoner’s Dilemma. Our results suggest that in large networks a minimum level of heterogeneity is necessary for a society to become evolutionary viable. Moreover, there is an optimal range of heterogeneity levels that maximize the resilience of the society facing an increasing number of social dilemmas. Finally, as the level of degree heterogeneity increases, the evolutionary dominance of either cooperators or defectors in a society increasingly depends on the initial state of a few influential individuals. Our findings imply that neither very unequal nor very equal societies offer the best evolutionary outcome.

## Introduction

It is well known that the structure of complex networks affects the outcomes of dynamical processes^1,2^, such as epidemic outbreaks^3–5^, diffusion of innovations^6,7^, opinion formation^8–10^ and behavioral evolution^11,12^. In that context, network heterogeneity is a topological feature of complex networks that is of particular importance^13–17^. The network heterogeneity refers to the heterogeneity among the number of ties maintained by each individual in a network; it is often measured by the variance of the degree distribution^18^ and called degree heterogeneity. Until now most works have, however, dealt with structures that exhibit opposite levels of degree heterogeneity, such as networks where all individuals have a similar number of network ties or networks with a power law distribution of the individuals’ ties. In this paper, we explore the gap between these two extremes in the context of evolutionary game theory^19,20^ and the problem of the evolution of cooperation^21,22^. Our goal is to understand if (1) there is a minimum threshold of heterogeneity necessary for the evolution of cooperation, and (2) if is there an optimal level of heterogeneity?

Previous theoretical works have extensively explored the role of the population structure in the evolution of cooperation. Yet, they have mainly dealt with structures that exhibit opposite levels of degree heterogeneity^23–34^. In this regard, regular and highly symmetric structures with low levels of heterogeneity–such as lattices^11,25–27^–have been shown to promote a stable co-existence dynamics between different competing behaviors, such as cooperators and defectors^35^. This co-existence dynamics stems from the formation of compact clusters that constrain the evolution of cooperation to the boundaries between regions dominated by individuals of a single type. In contrast, structures with high levels of degree heterogeneity (e.g. scale-free networks) prompt a coordination-like dynamics^36,37^ that result in the population being quickly driven to a monomorphic state which is dominated by one of the existing behaviors (*i*.*e*. either only cooperators or defectors). In these heterogeneous networks, the observed coordination dynamics results from the existence of higher degree nodes (i.e. hubs) that act as role models and lead to effective dynamics of competition between hubs. Thus, while both low and high heterogeneous networks promote cooperation, heterogeneous networks are able to promote cooperation in a wider range of social dilemmas.

Few works, though, have explored how a gradual interpolation between extreme levels of topological features in complex networks impact the outcome of dynamical processes. For instance, *Santos et al*.^38^ explored how the randomization of the connectivity patterns of a network–interpolating from a regular to a randomized homogeneous network–impacts the evolution of cooperative behavior and epidemic spreading. Following this line of research, we explore in this paper how the level of degree heterogeneity impacts the evolution of cooperation. For this purpose we analyze a wide range of networks with different levels of degree heterogeneity to show to which extent degree heterogeneity facilitates the dominance or co-existence of cooperators and/or defectors in a society.

Previous works have linked degree heterogeneity and degree variance also with social diversity, in the sense that it captures diversity among individual’s degrees of popularity and influence^18^. Social diversity is, however, not limited to popularity and influence, nor is it only able to be modeled through to the interaction structure of the population. Indeed, recent works have explored how diversity among interactions weights^39^, individuals’ aspirations^40^, wealth distribution^41–43^, age distribution^44^, and the type of social dilemmas faced by individuals^45^ play an important role in dictating the fate of cooperative behavior in social systems. Central for the positive impact of diversity in promoting cooperation is the heterogeneous distribution of wealth and influence it generates^46^. Thus our work also contributes to a better understanding of how social diversity drives the evolution of cooperation in complex networks.

To explore how different levels of degree heterogeneity impact the evolution of cooperation, we make extensive Monte Carlo simulations of the evolutionary dynamics. Moreover, we consider a wide range of populations whose structures smoothly interpolate between low and high levels of heterogeneity (see Model Section). Our findings show that many situations require a minimum threshold level of heterogeneity for cooperation to be sustainable. Moreover, there is an optimal range of heterogeneity that maximizes the population’s resilience across a wide range of social dilemmas. This is the case because an increasingly heterogeneous network structure becomes increasingly correlated with the initial state of a few highly influential individuals (see Discussion section). The findings imply that neither very equal nor extremely unequal societies, in terms of their level of degree heterogeneity, tend to offer the best evolutionary outcome.

## Model

Let us consider a population with *Z* individuals. The structure of interaction between the *Z* individuals of the population is modeled by means of a complex network, where nodes represent individuals, and the links between the nodes represent social relationships between the individuals. The total number of links of an individual *i* corresponds to his/her degree *z*_*i*_. The degree distribution, *D*(*z*), describes the fraction of individuals with degree *z*. To generate networks with different levels of degree heterogeneity *D*(*z*), we use an algorithm of network growth and biased preferential attachment by *Fortunato et al*.^47^. The algorithm proceeds as follows: it starts from a clique of three connected nodes and then sequentially *Z* − 3 other nodes are added; each of the newly added nodes gets attached to two pre-existing ones sampled proportionally to *t* ^*α*^ (*α* ≥ 0.0) where *t* corresponds to the age of the nodes. In the limiting case of *Z* → ∞, the networks exhibit a degree distribution of the form *D*(*z*) ≈ *z* ^−*γ*^ with *γ* = (1 + *α*)/*α* ^47,48^. Here *α* takes the role of the degree heterogeneity parameter exhibiting a one-to-one relationship with var(*z*), so that a low (large) *α* is associated with a low (large) level of degree variance or heterogeneity.

Let us also consider that individuals behave unconditionally either as *Cooperators* (**C**) or *Defectors* (**D**). At each moment, there are *k* **C**s and *Z* − *k* **D**s in the population. The evolutionary process follows the *fermi update rule* ^49,50^, thus at each time step a random individual (A) imitates the strategy of a random neighbor, B, with the probability

1 $${p}_{A\to B}=\frac{1}{1+{e}^{-\beta ({f}_{B}-{f}_{A})}}$$

where *β* regulates the selection pressure (*i*.*e*. the level of randomness of the individuals’ decision to be a cooperator or a defector), while *f*_*i*_ corresponds to the fitness of the individual *i*. When *β* ≪ 1, the population evolves under weak selection pressure; when *β* ≫ 1 the population evolves under strong selection pressure. In the limit of strong selection (large *β*), the results obtained under the *fermi update rule* become qualitatively similar to the replicator dynamics^51^.

Individuals obtain a payoff from each interaction with their neighbors. The value of the payoff depends only on the strategies of both. The four possible outcomes can be summarized in the following payoff matrix:


where *R* represents the Reward payoff resulting from mutual cooperation; *P* is the Punishment payoff from mutual defection; *S* is the Sucker’s payoff obtained by a Cooperator that faced a Defector; and *T* represents the Temptation payoff that a Defector obtains when facing a Cooperator. Here, we consider a simplified parameter space in which *R* = 1, *P* = 0, *T* = *λ* and *S* = 1 − *λ* with 1 ≤ *λ* ≤ 2. Hence, *λ* represents both the temptation for defection and the fear of being cheated, encapsulating the harshness of the dilemma faced by individuals.

The fitness of an individual corresponds to the accumulated payoff over all interactions in which he/she participates and can be computed as

2 $${f}_{i}={n}_{i}^{C}\mathrm{(1}+\lambda )-{s}_{i}\lambda ({n}_{i}^{D}+{n}_{i}^{C})$$

where ${n}_{i}^{C}$ (${n}_{i}^{D}$) is the number of **C**s (**D**s) in the neighborhood of *i* (${n}_{i}^{C}+{n}_{i}^{D}={z}_{i}$) and *s*_*i*_ = 1 (0) if *i* is a **C** (**D**). Moreover, *λ* defines the strength of the dilemma faced by individuals. When *λ* > 0, individuals engage in the *Prisoner’s Dilemma* (**PD**) and *λ* captures both the temptation to defect towards a **C** and the fear of being cheated by a **D**.

In order to measure the impact of different network structures on the evolution of cooperation, we measure the level of cooperation (*η*_*x*0_). More details on how the level of cooperation (*η*_*x*0_) is computed can be found in the Methods section. We estimate *η*_*x*0_ starting from a fraction *x*_0_ of cooperators and for different levels of selection pressure *β*, degree heterogeneity *α*, and harshness of the social dilemma *λ*. The level of cooperation (*η*_*x*0_) can thus be understood as the expected number of cooperators in the equilibrium. In order to explore the role that different individuals play in the evolutionary dynamics, we classify the individuals according to their network degree^18^. Hence let us denote nodes as low degree (**LDN**) if their degree is bounded by 3*z*_*i*_ ≤ *m*_*α*_; medium degree (**MDN**) when *m*_*α*_ < 3*z*_*i*_ ≤ 2*m*_*α*_; and high degree (**HDN**) for 3*z*_*i*_ > 2*m*_*α*_, where *m*_*α*_ corresponds to the observed maximum degree of an individual in a network with a level of degree heterogeneity of *α*.

## Results and Discussion

**Figure 1:**
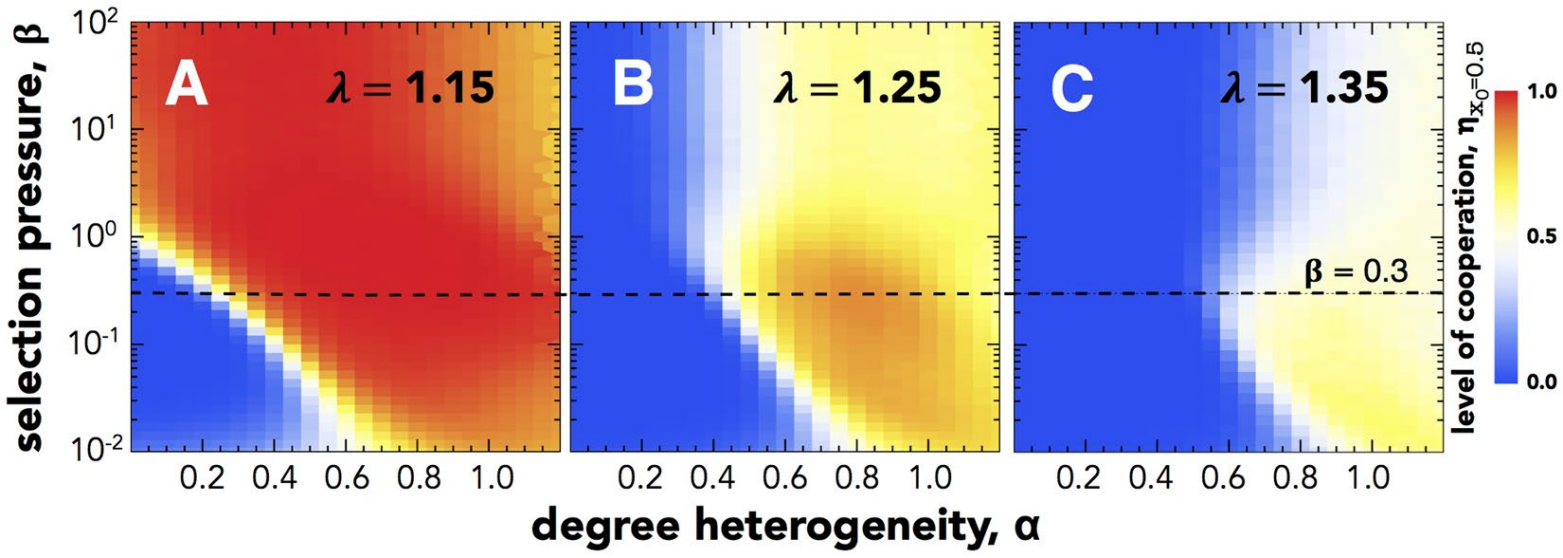
The impact of degree heterogeneity (0.0 ≤ *α* ≤ 1.25) and intensity of selection (10^−2^ ≤ *β* ≤ 10^2^) on the level of cooperation (${\eta }_{{x}_{0}\mathrm{=0.5}}$) under different values of the harshness of the social dilemmas that the individuals face: *λ* = 1.15 (left), 1.25 (center) and 1.35 (right). Blue areas indicate the parameters that lead to a full dominance of Defector and Red regions denote areas of Cooperator dominance. White areas correspond to scenarios in which it is equally likely for the population to reach either a state fully dominated by Cooperators or Defectors. The horizontal line marks *β* = 0.3. All simulations have been conducted in populations with *Z* = 10^3^ individuals and start from a configuration with equal composition of strategies.

We start by analyzing how the level of degree heterogeneity (*α*) and the selection pressure (*β*) impact the level of cooperation (${\eta }_{{x}_{0}}$) under different values of dilemma strength (*λ*). In the panels A, B and C of Fig. 1, three different regions can be highlighted according to their evolutionary outcome: (*i*) the domain of parameters strictly dominated by **D**s (blue region); (*ii*) the domain of parameters strictly dominated by **C**s (red region) and (*iii*) the domain of parameters where neither **C**s or **D**s are strictly dominant. The results in Fig. 1 suggest that cooperation requires a minimum level of degree heterogeneity (*α*) to be evolutionary viable – *i*.*e*. to achieve a state of **C** dominance with a non-zero likelihood. This result is particularly evident under weaker selection regimes (*β* < 1). Moreover, there is a region in the *α* × *β* domain under which the viability of cooperation is more resilient to variations in the harshness of the social dilemma faced by individuals (*λ*).

**Figure 2:**
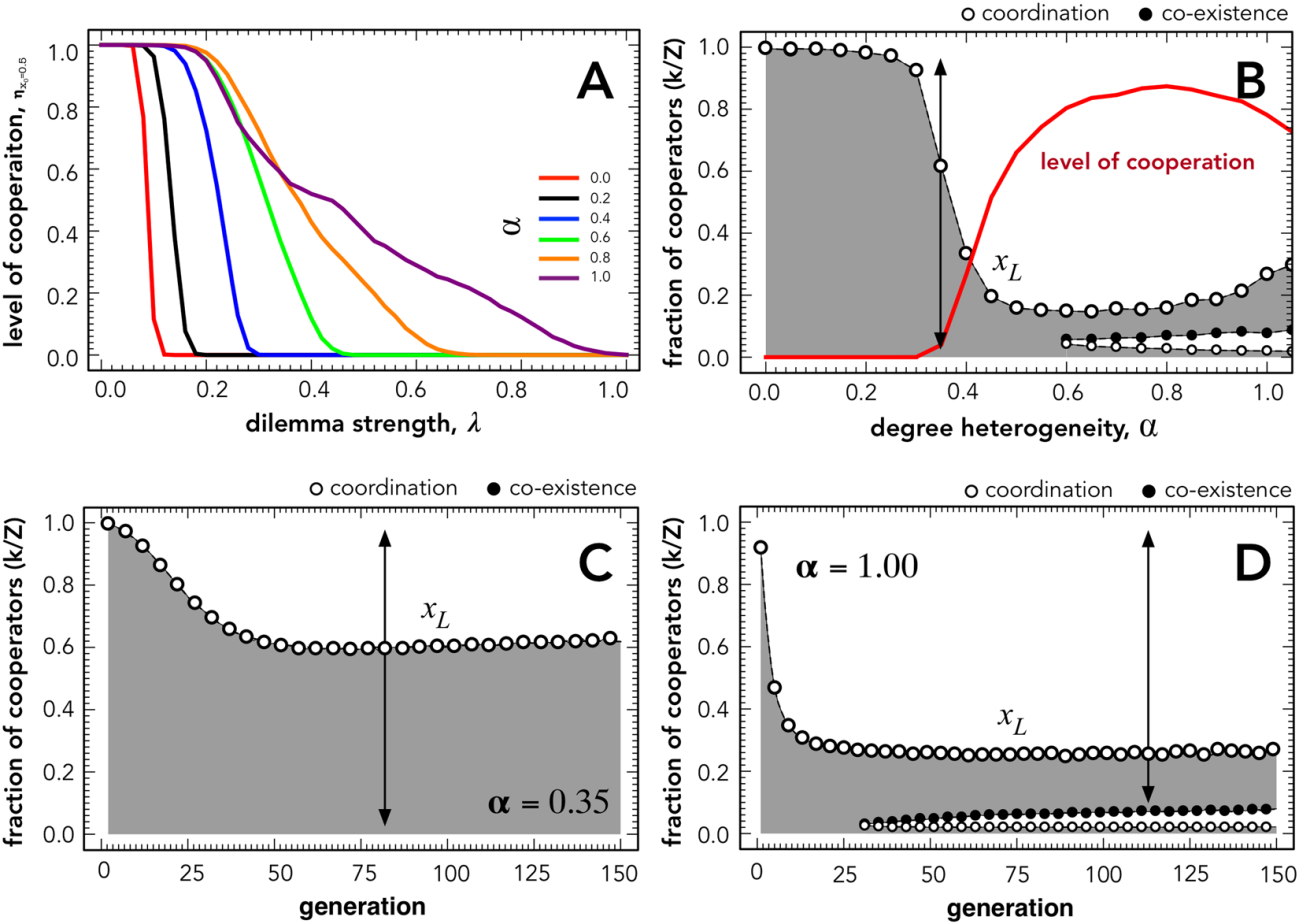
Population-wide dynamics for a wide range of levels of heterogeneity. (Panel A) shows how the level of cooperation changes for variation of *λ* and *α* with *β* = 0.3. Although the level of cooperation ${\eta }_{{x}_{0}}$ increases with increasing *α* the range of *λ* strictly dominated by cooperators decreases. (Panel B) shows the Average Gradient of Selection (Γ_*g*_(*k*)) after 150 generations for *λ* = 1.25 and *β* = 0.3 for a wide range of *α* values. The red line shows how the respective levels of cooperation change under the parameters as used to compute Γ_*g*_(*k*). Panels C and D show how Average Gradient of Selection changes over time until the 150^th^ generation. In (panels B, C and D), *λ* = 1.25 and *β* = 0.3. The dark areas denote regions in which Γ_*g*_(*k*) < 0 and white areas in which Γ_*g*_(*k*) > 0.

It must be noted that optimal levels of selection pressure have been identified in previous works^26,27,52^. Here, though, we show that this effect is restricted to a certain interval of network heterogeneity, in our case for *α* > 0.6. Figure 2A shows how the level of cooperation is affected by degree heterogeneity for different levels of the dilemma strength. While a sharp transition in the levels of cooperation characterizes the evolution under small values of degree heterogeneity, increasing values of degree heterogeneity lead to a slower decay of cooperation with rising levels of dilemma strengths.

Previous works have shown that heterogeneous networks facilitate a coordination-like dynamics at the population-wide level^36^. Hence, next we compute the Average Gradient of Selection (Γ_*g*_(*k*)) in order to inspect of the nature of the population-wide dynamics with different levels of degree heterogeneity. Details on how to compute Γ_*g*_(*k*) can be found in the methods section. In a nutshell, negative (positive) values of Γ_*g*_(*k*) indicate that a population with *k* Cooperators and *Z* − *k* Defectors population is more likely to see an decrease (increase) in the number of cooperators. For instance, in a well-mixed population (i.e. a complete graph, where all individuals are connected with each other) and when individuals engage in the Prisoner’s Dilemma, we observe Γ_*g*_(*k*) to be always negative within the interval 0 < *k* < *Z*. Moreover, Γ_*g*_(*k*) has two trivial solutions, one at *k* ^*^ = 0 and another at *k* ^*^ = *Z*. Hence, we inspect the existence of internal solutions (*k* ^*^) that lie inside the domain 0 < *k* ^*^ < *Z*.

Figure 2B shows the internal fixed points of the Average Gradient of Selection (Γ_*g*_(*k*)) at the 150^th^ generation. Gray areas represent regions in which Γ_*g*_(*k*) < 0 and white areas regions in which Γ_*g*_(*k*) > 0. Arrows point towards the direction of selection. White circles represent coordination points. These are unstable fixed points, because when the population is close to one of this points it will evolve away from such state. Conversely, black circles represent co-existence points. These are stable fixed points, because the population will converge to such a state. We see that for the interval of heterogeneity under analysis the Γ_*g*_(*k*) is mostly dominated by a single coordination point (*x*_*L*_), which implies that the population will be driven to a monomorphic state, depending on the initial fraction of cooperators (*x*_0_). Hence, these results reassure us that measuring the level of cooperation (${\eta }_{{x}_{0}}$) equates to finding the likelihood with which a population reaches a state of full cooperation.

Figure 2C and D shows the internal fixed points of Γ_*g*_(*k*) during the first 150 generations when *α* = 0.35 and *α* = 1.00, respectively. Although at the beginning the Γ_*g*_(*k*) is mostly a negative, the evolutionary dynamics will lead to the emergence of an internal coordination point, which shapes the outcome of the population.

**Figure 3:**
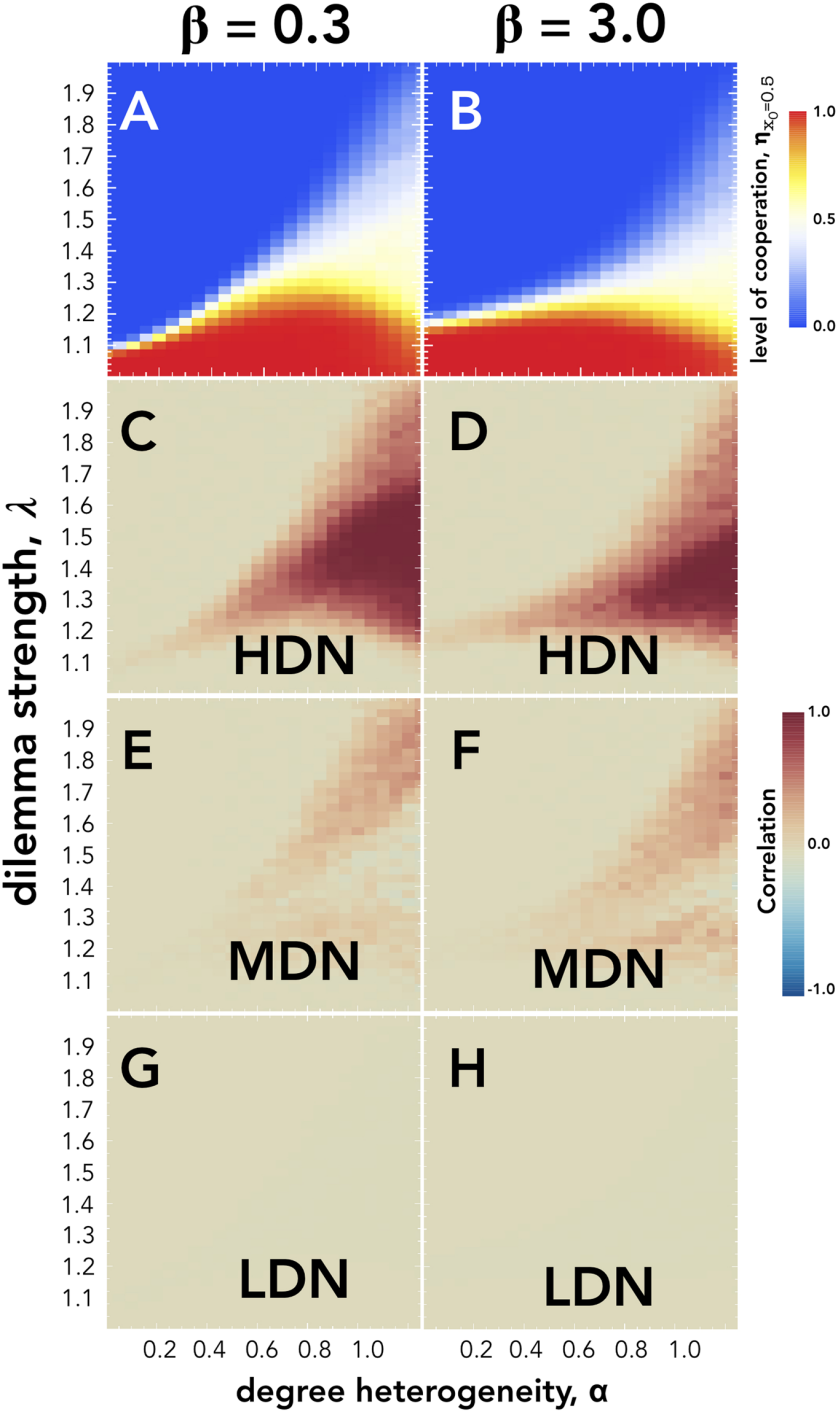
Panels A and B show the level of cooperation (*η*) while panels C, D, E and F show the correlations between the initial state of *Low Degree Nodes* (**LDN**), *Medium Degree Nodes* (**MDN**) and *High Degree Nodes* (**HDN**). All panels explore these quantities as a function of the harshness of the social dilemmas *λ* the individuals face and the degree heterogeneity *α* of the population structure. The left column shows results for *β* = 0.3 while the right column considers *β* = 3.0. All results have been computed for a population size of *Z* = 10^3^ and an average degree of 〈*k*〉 = 4.

Next, we show the level of cooperation (${\eta }_{{x}_{0}=0.5}$) as a function of the temptation to defect (*λ*) and of the level of degree heterogeneity (*α*) in the case of a weak (*β* = 0.3) and a strong (*β* = 3.0) selection pressure (see Fig. 3A and B). The level of heterogeneity largely defines the domain of social dilemmas under which cooperation is evolutionary viable. Although increasing levels of degree heterogeneity diminishes the range of *λ* that are strictly dominated by Ds, the degree heterogeneity also does not increase the region strictly dominated by **C**s. In fact, for low selection pressures there is an optimal level of heterogeneity that maximizes the range of social dilemmas strictly dominated by **C**s. For *β* = 0.3 this is between 0.6 ≤ *α* ≤ 0.9. This implies that if a population structure evolves to become increasingly heterogeneous, the population will eventually reach a level under which cooperation becomes less viable. Moreover, Fig. 3A and B show that for increasing levels of degree heterogeneity there is an increase in the region in which neither strategy is strictly dominant (white area), a result that is also suggested from the analysis of the *α* × *β* domain in Fig. 1A,B and C.

We scrutinize the mechanisms that lead to the prevalence of balanced levels of cooperation (white regions) for such a wide range of parameters. Our hypothesis is that the evolutionary outcome of the population becomes correlated with the initial state of the most well-connected individuals. With increasing levels of degree heterogeneity, the higher connected individuals (hubs) also receive more and more connections during the network growth. In consequence, the network becomes increasingly more unequal in terms of the distribution of influence and wealth. In other words, the fact that a small number of individuals in the population can participate in the majority of the available social interactions, thus accumulating a proportionally large fitness, results in these individuals dictating the outcome of the population according to their initial state.

To explore this hypothesis, we compute the phi correlation (*ϕ*_M_) between the initial strategy of individuals in a degree class (**LDN**, **MDN** or **HDG**) and the final state of the population. This quantity is formally defined as

3 $${\varphi }_{{\rm{M}}}=\frac{{n}_{{\rm{M}}}(C,C){n}_{{\rm{M}}}(D,D)-{n}_{{\rm{M}}}(D,C){n}_{{\rm{M}}}(D,C)}{\sqrt{{n}_{{\rm{M}}}(C,\bullet ){n}_{{\rm{M}}}(\bullet ,C){n}_{{\rm{M}}}(D,\bullet ){n}_{{\rm{M}}}(\bullet ,D)}}$$

where *n*_M_(*X*, *Y*) is the number of times we observe an individual in the degree class M with initial strategy *X* in a simulation where the population ended in a state dominated by strategy *Y*, *n*_M_(*X*, •) = *n*_M_(*X*, *Y*) + *n*_M_(*X*, *X*) and *n*_M_(•, *X*) = *n*_M_(*X*, *X*) + *n*_M_(*Y*, *X*). Hence, when *ϕ*_M_ = 0, the initial state of the nodes in degree class M are not correlated with the final state of the population, however when *ϕ*_M_ = 1, the population always ends up in a state dominated by the initial strategy of the nodes of degree class M. Conversely, when *ϕ*_M_ = −1, the population always ends up in the opposite state of the initial strategy of the nodes of degree class M.

**Figure 4:**
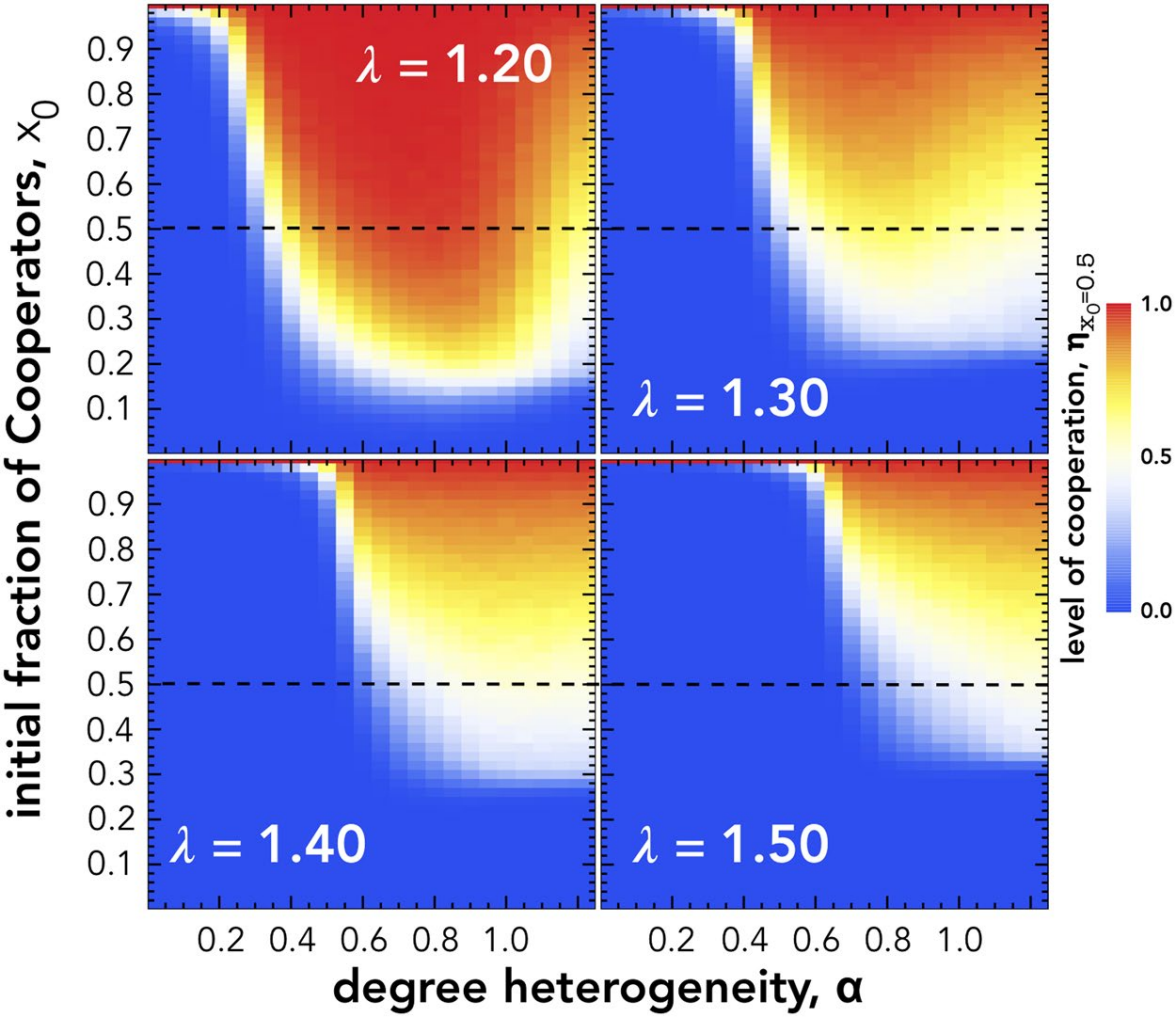
Level of Cooperation (${\eta }_{{x}_{0}}$) as a function of the initial fraction of Cooperators, and for different levels of degree heterogeneity, *α*. The red areas correspond to areas that are strictly dominated by Cooperators, the blue areas are dominated by Defectors. The horizontal line indicates *x*_0_ = 0.5. All simulations have been conducted with populations of *Z* = 10^3^ individuals, an average degree of 〈*z*〉 = 4 and a selection pressure of *β* = 0.3.

Figure 3C to H show the correlation between the initial state of individuals from different degree classes and the final state of the population. We draw three main conclusions from these results: (*i*) in the region dominated by a single strategy (blue and red areas in Fig. 4A and B) the final state is not predicted by the elements of any of the degree classes; (*ii*) Medium degree (**MDN**) and Low degree (**LDN**) nodes are weak predictors of the final level of cooperation; and (*iii*) the most connected, high degree, individuals (**HDN**) play a fundamental role in the region associated with a balanced levels of cooperation. In this region, we observe a strong correlation between their initial state and the fate of the population, meaning that the evolutionary outcome of the society depends on the probability (set by the initial fraction of cooperators parameter) of the highly connected individuals starting as cooperators or defectors. This confirms the hypothesis that the evolutionary outcome is driven by the initial state of the most influential individuals in the population. Moreover, **HDN** nodes play a central role in determining the final level of cooperation of the population in domains associated with the phase transition between the two strictly dominance regimes. Indeed, at this point the evolution of cooperation is dictated by the initial state of the most influential and wealthiest individuals.

However, the initial state of the most influential individuals (**HDN**) does not suffice in explaining what determines the final evolutionary outcome of the society. Without the existence of other **C**s in the population, the odds of cooperation being sustainable are very slim. Figure 4 shows how the evolutionary outcome (${\eta }_{{x}_{0}}$) depends on the initial abundance of cooperators (*x*_0_ in the *Y*-axis) under different heterogeneity levels (*α* in the *X*-axis) and the temptation to defect (*λ*). Again, it is clear that the evolution of cooperation requires a minimum level of degree heterogeneity and, moreover, a minimum number of cooperators to be evolutionary viable. For increasingly difficult social dilemmas, *i*.*e*. larger *λ*, cooperation viability requires a larger level of degree heterogeneity, hence a more unequal population.

**Figure 5:**
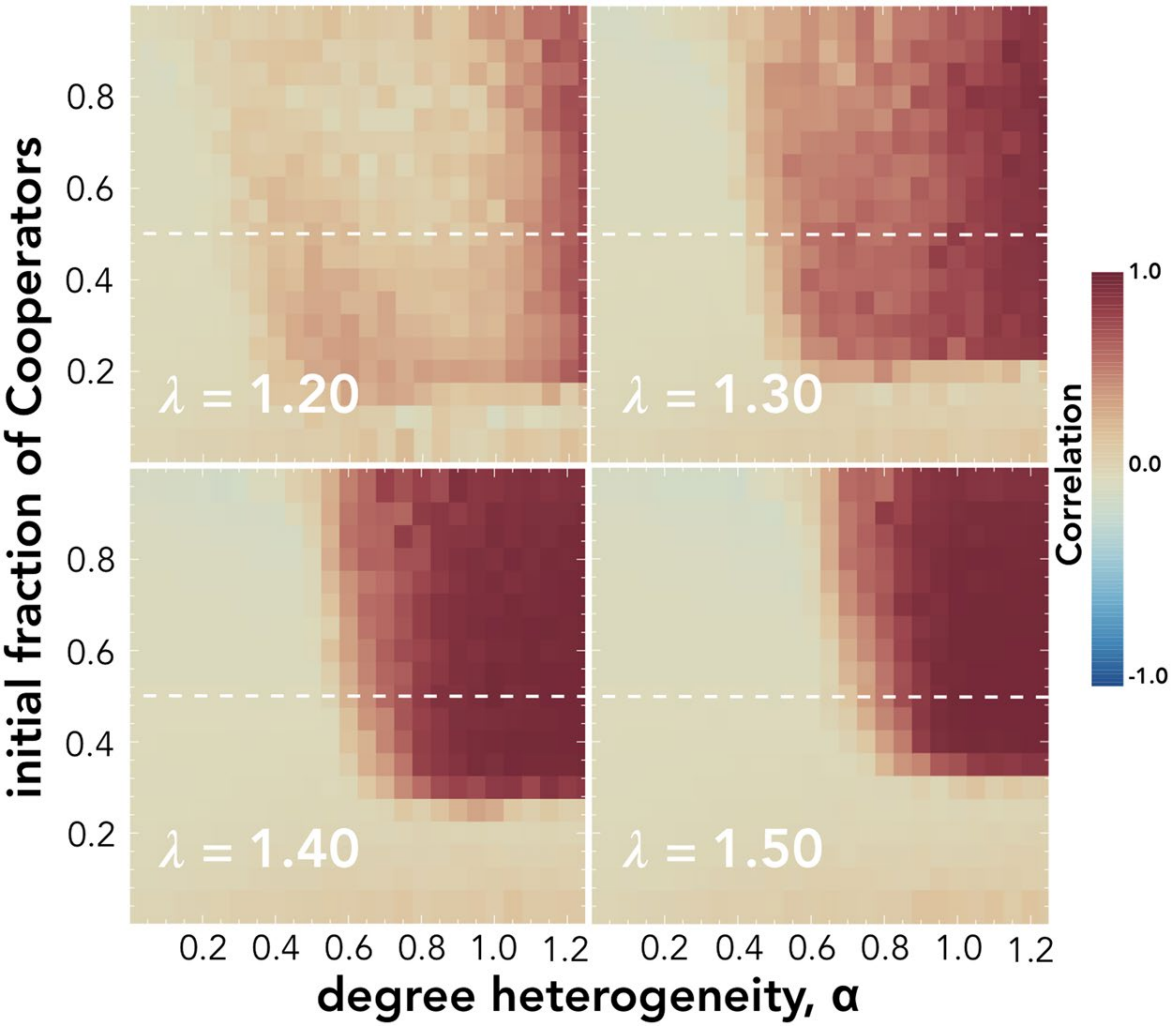
Correlation between initial state of high degree nodes and the final state of the population (*ϕ*_HDN_) as a function of the initial fraction of Cooperators and for different levels of degree heterogeneity (*α*). Red (Yellow) areas correspond to regions in which the initial state of higher degree nodes is highly correlated (uncorrelated) with the final state of the population. All simulations have been conducted with populations of *Z* = 10^3^ individuals, average degree of 〈*z*〉 = 4 and selection pressure of *β* = 0.3.

Figure 5 shows the correlation between the initial state of the most influential individuals (**HDN**) and the final state of the population under similar conditions as considered in Fig. 4. The obtained results suggest, once again, a strong correlation in the domain characterized by a balanced levels of cooperation (non-blue or non-red areas in Fig. 4).

## Conclusions

In this work we have explored how different levels of degree heterogeneity impact the evolution of cooperation in the Prisoner’s Dilemma. To that end, we made use of an algorithm of network growth and biased preferential attachment to generate a large set of network structures that interpolate between low and high levels of heterogeneity.

We find evidence of a threshold level of degree heterogeneity above which there is a substantial increase in the range of dilemmas where cooperation is evolutionary dominant. More importantly, we show that increasing levels of degree heterogeneity lead to an evolutionary trade off for populations: although the range of dilemmas in which cooperation can evolve increases, the range of dilemmas under which cooperation is strictly dominant decreases. Indeed, we observe that the evolution of cooperation becomes coupled with the initial state of the most well connected individuals. Hence, the ability of populations to overcome the underlying social dilemmas individuals engage becomes tied to the ability of a few individuals do so. Our findings imply that neither equal nor very unequal societies (in terms of degree heterogeneity) tend to offer the best evolutionary outcome, but instead suggest that there is an optimal level of equality. Although here we have explored static structures, recent works have shown how co-evolution of population structure often leads to an increase in degree heterogeneity^53–56^. Whenever the degree heterogeneity of a population increases, it also increases the dependence of the population on the decisions of a handful of influential individuals.

Open questions, though, are whether competition between societies can explain the preferential selection of heterogeneous social structures and whether a particular level of heterogeneity is favored under such a competitive environment. Finally, we explored the role of degree heterogeneity in the context of the evolution of cooperation. Our results suggest that there is a need to pay attention to the role that different levels of network heterogeneity play in the context of opinion dynamics and epidemic outbreaks.

## Methods

### Level of cooperation

(${\eta }_{{x}_{0}}$) is estimated by averaging the number of cooperators in the population after a large transient period of up to 10^5^ generations from 10^4^ independent simulations. Each simulation lasts for 5 × 10^3^ generations and starts from a fraction *x*_0_ of **C**s that are randomly distributed across the network. Given the coordination nature of the population-wide dynamics in heterogeneous networks^36^, ${\eta }_{{x}_{0}}$ provides a good approximation of the likelihood of a population reaching a monomorphic state dominated by **C**s. Along the manuscript we compute under ${\eta }_{{x}_{0}}$ different scenarios of degree heterogeneity (*α*), selection pressure (*β*) and the temptation to defect (*λ*).

### Average Gradient of Selection

(AGoS) captures the population-wide dynamics on structured populations. This quantity is the numerical counterpart of the drift term in a *Birth-Death* stochastic processes on finite and *well-mixed* populations^57^. We can estimate this quantity conveniently by computing the difference between the probability to increase (${\xi }_{g}^{+}(k)$) and the probability to decrease (${\xi }_{g}^{-}(k)$) the number of Cooperators by one when the population is in a state with *k* Cooperators and *Z* − *k* Defectors. The AGoS is, thus, formally defined as

4 $${{\rm{\Gamma }}}_{g}(k)={\xi }_{g}^{+}(k)-{\xi }_{g}^{-}(k)$$

where ${\xi }_{g}^{\pm }(k)$ is numerically computed according to

5 $${\xi }_{g}^{\pm }(k)=\frac{1}{{{\rm{\Lambda }}}_{g}(k)}\sum _{\omega =1}^{{\rm{\Omega }}}\sum _{t=1}^{{t}_{max}}\,\delta (k,{k}_{t}){\rm{\Theta }}(\frac{t}{Z},g)){\xi }_{\omega }^{\pm }(k,t)$$

where Λ_*g*_(*k*) accounts for the total number of times the population was observed in a state with *k* Cooperators at generation *g* over all Ω simulations and Θ(*a*,*b*) is a square discrete function that is equal to 1 if *b* − 1 ≤ *a* < *b*, being 0 otherwise. Finally ${\xi }_{\omega }^{\pm }(k,t)$ is the transition probability at time *t* of time-series *ω*, that is

6 $${\xi }_{\omega }^{\pm }(k,t)=\frac{1}{Z}\sum _{i=1}^{Z}\,\frac{1}{{z}_{i}}\sum _{j\in {\zeta }_{i}}\,\frac{1-\delta ({s}_{j},{s}_{i})}{1+{e}^{-\beta ({f}_{j}\mp {f}_{i})}}$$

where *ζ*_*j*_ is the set of closest neighbours of *j* and *k*_*t*_ is the number of **C**s at time *t*. In this expression, the first sum is calculated over each individual in the population and the second sum is calculated over each individual’s neighbourhood. In order to estimate Γ(*k*) we let a population evolve for 150 generations. Each simulation of the evolutionary process starts from a possible random state (i.e. starting from a random number of Cooperators *k* randomly selected from the interval 1 < *k* < *Z*). We repeat this for a total of Ω = 2.5 × 10^7^ times. For each iteration we estimate the ${\xi }_{\omega }^{\pm }(k,t)$ as described above.
